# A genome-wide gene-environment interaction study of breast cancer risk for women of European ancestry

**DOI:** 10.1186/s13058-023-01691-8

**Published:** 2023-08-09

**Authors:** Pooja Middha, Xiaoliang Wang, Sabine Behrens, Manjeet K. Bolla, Qin Wang, Joe Dennis, Kyriaki Michailidou, Thomas U. Ahearn, Irene L. Andrulis, Hoda Anton-Culver, Volker Arndt, Kristan J. Aronson, Paul L. Auer, Annelie Augustinsson, Thaïs Baert, Laura E. Beane Freeman, Heiko Becher, Matthias W. Beckmann, Javier Benitez, Stig E. Bojesen, Hiltrud Brauch, Hermann Brenner, Angela Brooks-Wilson, Daniele Campa, Federico Canzian, Angel Carracedo, Jose E. Castelao, Stephen J. Chanock, Georgia Chenevix-Trench, CTS Consortium, Emilie Cordina-Duverger, Fergus J. Couch, Angela Cox, Simon S. Cross, Kamila Czene, Laure Dossus, Pierre-Antoine Dugué, A. Heather Eliassen, Mikael Eriksson, D. Gareth Evans, Peter A. Fasching, Jonine D. Figueroa, Olivia Fletcher, Henrik Flyger, Marike Gabrielson, Manuela Gago-Dominguez, Graham G. Giles, Anna González-Neira, Felix Grassmann, Anne Grundy, Pascal Guénel, Christopher A. Haiman, Niclas Håkansson, Per Hall, Ute Hamann, Susan E. Hankinson, Elaine F. Harkness, Bernd Holleczek, Reiner Hoppe, John L. Hopper, Richard S. Houlston, Anthony Howell, David J. Hunter, Christian Ingvar, ABCTB Investigators, kConFab Investigators, Karolin Isaksson, Helena Jernström, Esther M. John, Michael E. Jones, Rudolf Kaaks, Renske Keeman, Cari M. Kitahara, Yon-Dschun Ko, Stella Koutros, Allison W. Kurian, James V. Lacey, Diether Lambrechts, Nicole L. Larson, Susanna Larsson, Loic Le Marchand, Flavio Lejbkowicz, Shuai Li, Martha Linet, Jolanta Lissowska, Maria Elena Martinez, Tabea Maurer, Anna Marie Mulligan, Claire Mulot, Rachel A. Murphy, William G. Newman, Sune F. Nielsen, Børge G. Nordestgaard, Aaron Norman, Katie M. O’Brien, Janet E. Olson, Alpa V. Patel, Ross Prentice, Erika Rees-Punia, Gad Rennert, Valerie Rhenius, Kathryn J. Ruddy, Dale P. Sandler, Christopher G. Scott, Mitul Shah, Xiao-Ou Shu, Ann Smeets, Melissa C. Southey, Jennifer Stone, Rulla M. Tamimi, Jack A. Taylor, Lauren R. Teras, Katarzyna Tomczyk, Melissa A. Troester, Thérèse Truong, Celine M. Vachon, Sophia S. Wang, Clarice R. Weinberg, Hans Wildiers, Walter Willett, Stacey J. Winham, Alicja Wolk, Xiaohong R. Yang, M. Pilar Zamora, Wei Zheng, Argyrios Ziogas, Alison M. Dunning, Paul D. P. Pharoah, Montserrat García-Closas, Marjanka K. Schmidt, Peter Kraft, Roger L. Milne, Sara Lindström, Douglas F. Easton, Jenny Chang-Claude

**Affiliations:** 1 Division of Cancer Epidemiology, German Cancer Research Center (DKFZ), Heidelberg, Germany; 2 Department of Epidemiology, University of Washington School of Public Health, Seattle, WA USA; 3 Public Health Sciences Division, Fred Hutchinson Cancer Research Center, Seattle, WA USA; 4 Department of Public Health and Primary Care, Centre for Cancer Genetic Epidemiology, University of Cambridge, Cambridge, UK; 5 Biostatistics Unit, The Cyprus Institute of Neurology and Genetics, Nicosia, Cyprus; 6 Division of Cancer Epidemiology and Genetics, Department of Health and Human Services, National Cancer Institute, National Institutes of Health, Bethesda, MD USA; 7 Fred A. Litwin Center for Cancer Genetics, Lunenfeld-Tanenbaum Research Institute of Mount Sinai Hospital, Toronto, ON Canada; 8 Department of Molecular Genetics, University of Toronto, Toronto, ON Canada; 9 Department of Medicine, Genetic Epidemiology Research Institute, University of California Irvine, Irvine, CA USA; 10 Division of Clinical Epidemiology and Aging Research, German Cancer Research Center (DKFZ), Heidelberg, Germany; 11 Department of Public Health Sciences, and Cancer Research Institute, Queen’s University, Kingston, ON Canada; 12 Division of Biostatistics, Institute for Health and Equity, and Cancer Center, Medical College of Wisconsin, Milwaukee, WI USA; 13 Oncology, Clinical Sciences in Lund, Lund University, Lund, Sweden; 14 Department of Oncology, Leuven Multidisciplinary Breast Center, Leuven Cancer Institute, University Hospitals Leuven, Leuven, Belgium; 15 Institute of Medical Biometry and Epidemiology, University Medical Center Hamburg-Eppendorf, Hamburg, Germany; 16 Department of Gynecology and Obstetrics, Comprehensive Cancer Center Erlangen-EMN, University Hospital Erlangen, Friedrich-Alexander University Erlangen-Nuremberg, Erlangen, Germany; 17 Human Genetics Group, Spanish National Cancer Research Centre (CNIO), Madrid, Spain; 18 Centre for Biomedical Network Research on Rare Diseases (CIBERER), Instituto de Salud Carlos III, Madrid, Spain; 19 Copenhagen General Population Study, Herlev and Gentofte Hospital, Copenhagen University Hospital, Herlev, Denmark; 20 Department of Clinical Biochemistry, Herlev and Gentofte Hospital, Copenhagen University Hospital, Herlev, Denmark; 21 Faculty of Health and Medical Sciences, University of Copenhagen, Copenhagen, Denmark; 22 Dr. Margarete Fischer-Bosch-Institute of Clinical Pharmacology, Stuttgart, Germany; 23 iFIT-Cluster of Excellence, University of Tübingen, Tübingen, Germany; 24 German Cancer Consortium (DKTK) and German Cancer Research Center (DKFZ), Partner Site Tübingen, Tübingen, Germany; 25 Division of Preventive Oncology, German Cancer Research Center (DKFZ) and National Center for Tumor Diseases (NCT), Heidelberg, Germany; 26 German Cancer Consortium (DKTK), German Cancer Research Center (DKFZ), Heidelberg, Germany; 27 Canada’s Michael Smith Genome Sciences Centre, BC Cancer, Vancouver, BC Canada; 28 Department of Biology, University of Pisa, Pisa, Italy; 29 Genomic Epidemiology Group, German Cancer Research Center (DKFZ), Heidelberg, Germany; 30 Genomic Medicine Group, International Cancer Genetics and Epidemiology Group, Fundación Pública Galega de Medicina Xenómica, Instituto de Investigación Sanitaria de Santiago de Compostela (IDIS), Complejo Hospitalario Universitario de Santiago, SERGAS, Santiago de Compostela, Spain; 31 Grupo de Medicina Xenómica, Centro de Investigación en Red de Enfermedades Raras (CIBERER) y Centro Nacional de Genotipado (CEGEN-PRB2), Universidad de Santiago de Compostela, Santiago de Compostela, Spain; 32 Oncology and Genetics Unit, Instituto de Investigación Sanitaria Galicia Sur (IISGS), Xerencia de Xestion Integrada de Vigo-SERGAS, Vigo, Spain; 33 Department of Genetics and Computational Biology, QIMR Berghofer Medical Research Institute, Brisbane, QLD Australia; 34 Department of Computational and Quantitative Medicine, City of Hope, Duarte, CA USA; 35 City of Hope Comprehensive Cancer Center, City of Hope, Duarte, CA USA; 36 Team ‘Exposome and Heredity’, CESP, Gustave Roussy, INSERM, University Paris-Saclay, UVSQ, Villejuif, France; 37 Department of Laboratory Medicine and Pathology, Mayo Clinic, Rochester, MN USA; 38 Department of Oncology and Metabolism, Sheffield Institute for Nucleic Acids (SInFoNiA), University of Sheffield, Sheffield, UK; 39 Academic Unit of Pathology, Department of Neuroscience, University of Sheffield, Sheffield, UK; 40 Department of Medical Epidemiology and Biostatistics, Karolinska Institutet, Stockholm, Sweden; 41 Nutrition and Metabolism Section, International Agency for Research on Cancer (IARC-WHO), Lyon, France; 42 Precision Medicine, School of Clinical Sciences at Monash Health, Monash University, Clayton, VIC Australia; 43 Cancer Epidemiology Division, Cancer Council Victoria, Melbourne, VIC Australia; 44 Channing Division of Network Medicine, Department of Medicine, Brigham and Women’s Hospital and Harvard Medical School, Boston, MA USA; 45 Department of Epidemiology, Harvard T.H. Chan School of Public Health, Boston, MA USA; 46 Department of Nutrition, Harvard T.H. Chan School of Public Health, Boston, MA USA; 47 Division of Evolution and Genomic Sciences, Faculty of Biology, Medicine and Health, Manchester Academic Health Science Centre, School of Biological Sciences, University of Manchester, Manchester, UK; 48 North West Genomics Laboratory Hub, Manchester Centre for Genomic Medicine, Manchester Academic Health Science Centre, St Mary’s Hospital, Manchester University NHS Foundation Trust, Manchester, UK; 49 Usher Institute of Population Health Sciences and Informatics, The University of Edinburgh, Edinburgh, UK; 50 Cancer Research UK Edinburgh Centre, The University of Edinburgh, Edinburgh, UK; 51 The Breast Cancer Now Toby Robins Research Centre, The Institute of Cancer Research, London, UK; 52 Department of Breast Surgery, Herlev and Gentofte Hospital, Copenhagen University Hospital, Herlev, Denmark; 53 Centre for Epidemiology and Biostatistics, Melbourne School of Population and Global Health, The University of Melbourne, Melbourne, VIC Australia; 54 Human Cancer Genetics Programme, Spanish National Cancer Research Centre (CNIO), Madrid, Spain; 55 Institute for Clinical Research and Systems Medicine, Health and Medical University, Potsdam, Germany; 56 Department of Public Health Sciences, Queen’s University, Kingston, ON Canada; 57 Department of Preventive Medicine, Keck School of Medicine, University of Southern California, Los Angeles, CA USA; 58 Institute of Environmental Medicine, Karolinska Institutet, Stockholm, Sweden; 59 Department of Oncology, Södersjukhuset, Stockholm, Sweden; 60 Molecular Genetics of Breast Cancer, German Cancer Research Center (DKFZ), Heidelberg, Germany; 61 Department of Biostatistics and Epidemiology, University of Massachusetts, Amherst, Amherst, MA USA; 62 Division of Informatics, Imaging and Data Sciences, Faculty of Biology, Medicine and Health, Manchester Academic Health Science Centre, University of Manchester, Manchester, UK; 63 Nightingale and Genesis Prevention Centre, Wythenshawe Hospital, Manchester University NHS Foundation Trust, Manchester, UK; 64 NIHR Manchester Biomedical Research Unit, Manchester Academic Health Science Centre, Manchester University NHS Foundation Trust, Manchester, UK; 65 Saarland Cancer Registry, Saarbrücken, Germany; 66 University of Tübingen, Tübingen, Germany; 67 Division of Genetics and Epidemiology, The Institute of Cancer Research, London, UK; 68 Division of Cancer Sciences, University of Manchester, Manchester, UK; 69 Nuffield Department of Population Health, University of Oxford, Oxford, UK; 70 Surgery, Clinical Sciences in Lund, Lund University, Lund, Sweden; 71 Australian Breast Cancer Tissue Bank, Westmead Institute for Medical Research, University of Sydney, Sydney, NSW Australia; 72 Research Department, Peter MacCallum Cancer Center, Melbourne, VIC Australia; 73 Sir Peter MacCallum Department of Oncology, The University of Melbourne, Melbourne, VIC Australia; 74 Department of Surgery, Kristianstad Hospital, Kristianstad, Sweden; 75 Department of Epidemiology and Population Health, Stanford University School of Medicine, Stanford, CA USA; 76 Division of Oncology, Department of Medicine, Stanford Cancer Institute, Stanford University School of Medicine, Stanford, CA USA; 77 Division of Molecular Pathology, The Netherlands Cancer Institute, Amsterdam, The Netherlands; 78 Radiation Epidemiology Branch, Division of Cancer Epidemiology and Genetics, National Cancer Institute, Bethesda, MD USA; 79 Department of Internal Medicine, Johanniter GmbH Bonn, Johanniter Krankenhaus, Bonn, Germany; 80 Laboratory for Translational Genetics, Department of Human Genetics, KU Leuven, Leuven, Belgium; 81 VIB Center for Cancer Biology, VIB, Leuven, Belgium; 82 Department of Quantitative Health Sciences, Division of Epidemiology, Mayo Clinic, Rochester, MN USA; 83 Department of Surgical Sciences, Uppsala University, Uppsala, Sweden; 84 Epidemiology Program, University of Hawaii Cancer Center, Honolulu, HI USA; 85 Clalit National Cancer Control Center, Carmel Medical Center and Technion Faculty of Medicine, Haifa, Israel; 86 Department of Cancer Epidemiology and Prevention, M. Sklodowska-Curie National Research Oncology Institute, Warsaw, Poland; 87 Moores Cancer Center and Herbert Wertheim School of Public Health and Human Longevity Science, University of California, San Diego, La Jolla, CA USA; 88 Cancer Epidemiology Group, University Cancer Center Hamburg (UCCH), University Medical Center Hamburg-Eppendorf, Hamburg, Germany; 89 Department of Laboratory Medicine and Pathobiology, University of Toronto, Toronto, ON Canada; 90 Laboratory Medicine Program, University Health Network, Toronto, ON Canada; 91 INSERM UMR-S1138. CRB EPIGENETEC, Université Paris Cité, Paris, France; 92 School of Population and Public Health, University of British Columbia, Vancouver, BC Canada; 93 Cancer Control Research, BC Cancer, Vancouver, BC Canada; 94 Epidemiology Branch, National Institute of Environmental Health Sciences, NIH, Research Triangle Park, NC USA; 95 Department of Population Science, American Cancer Society, Atlanta, GA USA; 96 Cancer Prevention Program, Fred Hutchinson Cancer Research Center, Seattle, WA USA; 97 Department of Oncology, Centre for Cancer Genetic Epidemiology, University of Cambridge, Cambridge, UK; 98 Department of Oncology, Mayo Clinic, Rochester, MN USA; 99 Department of Quantitative Health Sciences, Division of Clinical Trials and Biostatistics, Mayo Clinic, Rochester, MN USA; 100 Division of Epidemiology, Department of Medicine, Vanderbilt Epidemiology Center, Vanderbilt-Ingram Cancer Center, Vanderbilt University School of Medicine, Nashville, TN USA; 101 Department of Surgical Oncology, University Hospitals Leuven, Leuven, Belgium; 102 Department of Clinical Pathology, The University of Melbourne, Melbourne, VIC Australia; 103 Genetic Epidemiology Group, School of Population and Global Health, University of Western Australia, Perth, WA Australia; 104 Department of Population Health Sciences, Weill Cornell Medicine, New York, NY USA; 105 Epigenetic and Stem Cell Biology Laboratory, National Institute of Environmental Health Sciences, NIH, Research Triangle Park, NC USA; 106 Department of Epidemiology, Gillings School of Global Public Health and UNC Lineberger Comprehensive Cancer Center, University of North Carolina at Chapel Hill, Chapel Hill, NC USA; 107 Biostatistics and Computational Biology Branch, National Institute of Environmental Health Sciences, NIH, Research Triangle Park, NC USA; 108 Division of Computational Biology, Department of Quantitative Health Sciences, Mayo Clinic, Rochester, MN USA; 109 Servicio de Oncología Médica, Hospital Universitario La Paz, Madrid, Spain; 110 Division of Psychosocial Research and Epidemiology, The Netherlands Cancer Institute - Antoni Van Leeuwenhoek Hospital, Amsterdam, the Netherlands; 111 Program in Genetic Epidemiology and Statistical Genetics, Harvard T.H. Chan School of Public Health, Boston, MA USA

**Keywords:** Breast cancer, Gene-environment interactions, Genetic epidemiology, European ancestry

## Abstract

**Background:**

Genome-wide studies of gene–environment interactions (G×E) may identify variants associated with disease risk in conjunction with lifestyle/environmental exposures. We conducted a genome-wide G×E analysis of ~ 7.6 million common variants and seven lifestyle/environmental risk factors for breast cancer risk overall and for estrogen receptor positive (ER +) breast cancer.

**Methods:**

Analyses were conducted using 72,285 breast cancer cases and 80,354 controls of European ancestry from the Breast Cancer Association Consortium. Gene–environment interactions were evaluated using standard unconditional logistic regression models and likelihood ratio tests for breast cancer risk overall and for ER + breast cancer. Bayesian False Discovery Probability was employed to assess the noteworthiness of each SNP-risk factor pairs.

**Results:**

Assuming a 1 × 10^–5^ prior probability of a true association for each SNP-risk factor pairs and a Bayesian False Discovery Probability < 15%, we identified two independent SNP-risk factor pairs: rs80018847(9p13)-*LINGO2* and adult height in association with overall breast cancer risk (OR_int_ = 0.94, 95% CI 0.92–0.96), and rs4770552(13q12)-*SPATA13* and age at menarche for ER + breast cancer risk (OR_int_ = 0.91, 95% CI 0.88–0.94).

**Conclusions:**

Overall, the contribution of G×E interactions to the heritability of breast cancer is very small. At the population level, multiplicative G×E interactions do not make an important contribution to risk prediction in breast cancer.

**Supplementary Information:**

The online version contains supplementary material available at 10.1186/s13058-023-01691-8.

## Background

Breast cancer is a complex disease involving interplay between lifestyle/environmental and genetic risk factors. Risk factors such as parity, breastfeeding, age at menarche, age at first full-term pregnancy, body mass index (BMI), height, mammographic density, exogenous hormonal use, and alcohol consumption are well-established [1–7]. Through continued collaborative efforts such as the Collaborative Oncological Gene-environment Study (COGS) and the OncoArray project [8], more than 200 common single nucleotide polymorphisms (SNPs) associated with risk of breast cancer have been identified [9–11].

Traditional genome-wide association study (GWAS) analyses assess the marginal effects of variants and might miss variants which only show an effect within certain strata in the population. These potential gene–environment interactions where SNPs are associated with disease risk in conjunction with lifestyle/environmental risk factors can be investigated through genome-wide gene-environment interaction studies (GEWIS) [12–15].

Very few genome-wide studies of gene-environment (G×E) interactions in breast cancer have been conducted to date, and three focused on the use of menopausal hormonal therapy as the single environmental risk factor [16–18]. An exploratory analysis of G×E interactions examined ten environmental risk factors and 71,527 SNPs selected from prior evidence, using data from approximately 35,000 cases and controls in the Breast Cancer Association Consortium (BCAC). That study identified two potential G×E interactions associated with breast cancer risk [19]. In the present study, we performed a comprehensive genome-wide analysis of gene–environment interactions for risk of overall breast cancer, as well as estrogen receptor positive (ER +) breast cancer using data from 72,285 cases and 80,354 controls participating in the BCAC.

## Methods

### Study sample

Analyses were conducted using data from 46 studies (16 prospective cohorts, 14 population-based case–control studies, and 16 non-population based studies) participating in the BCAC. We excluded participants if they were genotypically male, of non-European descent, or had a breast tumor of unknown invasiveness or in-situ breast cancer. Women with prevalent breast cancer at the time of recruitment or with unknown reference age (defined as age at diagnosis for cases and age at interview for controls) were also excluded from the analyses. Further, studies with fewer than 150 cases and 150 controls for the risk factor under evaluation were excluded from those analyses. Each participating study obtained informed consent from the participants and was approved by their local ethics committee.

### Risk factor data

Risk factor data from individual studies was checked for quality using a multi-step harmonization process based on a common data dictionary. Time-dependent risk factor variables were derived with respect to the reference date defined as date of diagnosis for cases and date of interview for controls. Analyses were conducted with the following risk factors among all women: age at menarche (per 2 years), parity (per 1 birth), adult height (per 5 cm), ever use of oral contraceptives (yes/no), and current smoking (yes/no). The analysis of age at first full-term pregnancy (per 5 years) was conducted among parous women only, and that of body mass index (BMI, per 5 kg/m^2^) was conducted among postmenopausal women only. Menopausal status was either self-reported or assigned as postmenopausal if the reference age was greater than 54 years.

### Genetic data

All samples were genotyped either using the iCOGS [20, 21] or OncoArray [9, 10, 22]. Briefly, iCOGS is a customized iSelect SNP genotyping array, consisting of ~ 211,000 SNPs [20, 21], whereas OncoArray includes ~ 533,000 SNPs of which nearly 260,000 were selected as a GWAS backbone (Illumina HumanCore) [22]. Detailed information is provided elsewhere [9, 10, 20–22]. Data were imputed to the 1000 Genomes Reference Panel (phase 3 version 5). Overall, 28,176 cases and 32,209 controls of European ancestry who were genotyped by the iCOGS array, and 44,109 cases and 48,145 controls who were genotyped using the OncoArray array were included in this analysis.

Genetic variants with imputation quality score < 0.5 in iCOGS or < 0.8 in OncoArray, or with minor allele frequency < 0.01, were excluded from the analyses. Variants in known breast cancer regions were also excluded from the analysis since interactions between known susceptibility variants and risk factors have been explored previously [23, 24]. After applying all exclusions, 7,672,870 genetic variants (SNPs and indels) were included in the analysis.

### Statistical analysis

Unconditional logistic regression was employed to assess the associations of SNPs and risk factors with breast cancer risk. Genotypes were assessed using the expected number of copies of the alternative allele (‘dosage’) as the covariate under a log-additive model. Interactions between genetic variants and risk factors were tested by comparing the fit of logistic regression models with and without an interaction term using likelihood ratio tests. All models were adjusted for reference age, study, and ten ancestry-informative principal components. To account for potential differential main effects of risk factors by study design, all models included an interaction term between risk factor and an indicator variable for study design (population-based vs. non-population based). Analyses with current smoking were further adjusted for former smoking.

Analyses were performed separately for overall and ER + breast cancer risk, and also separately by genotyping array. Array-specific results were combined using METAL [25]. Quantile–quantile (Q-Q) plots were assessed to examine the consistency of the distribution of p-values with the null distribution. Interaction P value less than 5E-07 was considered suggestive evidence of interaction. We also calculated Bayesian False Discovery Probabilities (BFDP) for all suggestive interactions, assuming a 1 × 10^–5^ prior probability of a true association for each SNP-risk factor pair. Overall, G×E interactions with BFDP < 15% were considered noteworthy [26]. For noteworthy SNP-risk pairs, we evaluated the G×E interaction also for ER-negative breast cancer risk. For noteworthy interactions, we conducted stratified analyses by categories of the risk factor. All analyses were conducted using R version 3.5.1.

We estimated the overall genome-wide contribution of G×E associations for each risk factor to the familial relative risk of breast cancer using LD score regression [27]. The analysis used the G×E interaction summary statistics and was restricted to HapMap3 SNPs with MAF > 5% in European population from the 1000 Genomes Project. Under the log-additive model, the G×E heritability on the frailty scale can be estimated by *h*_*f*_^*2*^ = *h*_*obs*_^*2*^ × *var(X)/P(1-P)*, where h_obs_^2^ is the observed heritability given by LD score regression, *var(X)* is the variance of the risk factor under evaluation, and *P* is the proportion of cases in the sample. The proportion of the familial relative risk (FRR) of breast cancer due to G×E interactions is then given by *h*_*f*_^*2*^/2log(λ) where λ is the familial relative risk to first degree relatives of cases (assumed to be 2) [28].

## Results

Studies included in the analysis are summarized in Additional file 1: Table S1. The number of cases and controls in each analysis varied from 61,617 cases and 74,698 controls for parity to 48,276 cases and 60,587 controls for current smoking (Additional file 1: Table S2). Consistent with the literature, increasing age at first full-term pregnancy, higher adult height, ever use of oral contraceptives, and current smoking were associated with increased overall breast cancer risk, whereas increasing age at menarche, being parous, increasing number of full-term pregnancies, and breast feeding were associated with decreased breast cancer risk (Additional file 1: Table S3).

**Fig. 1:**
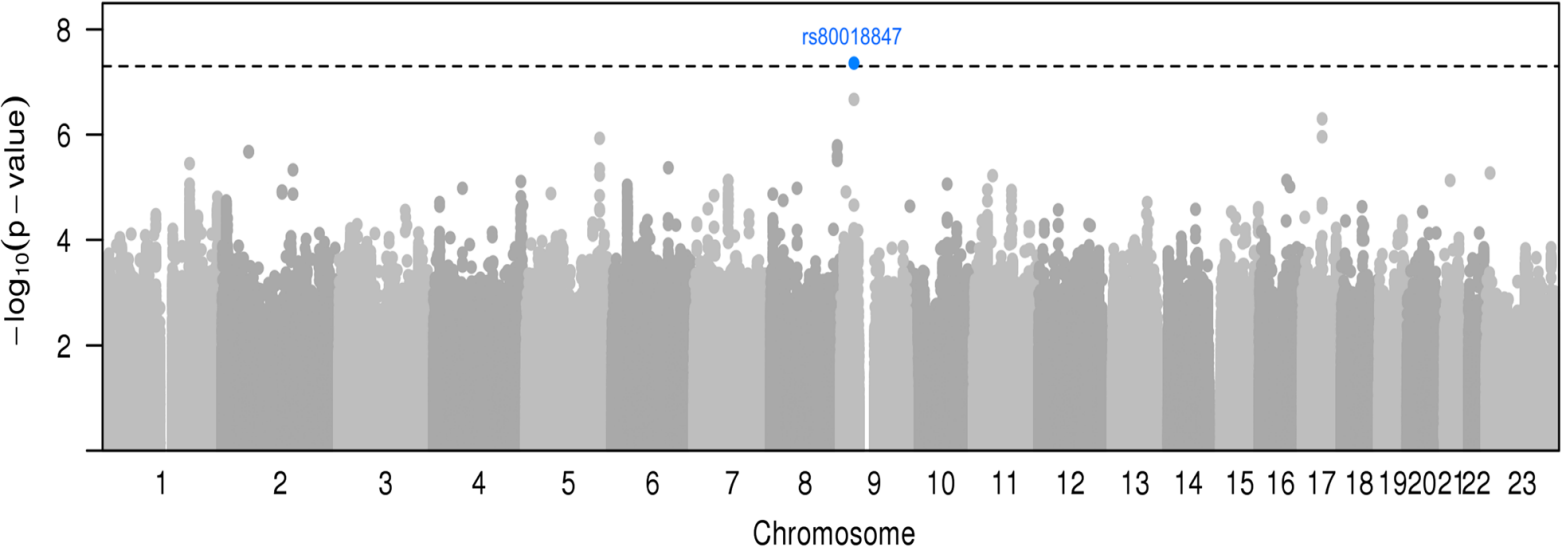
Manhattan plot of genome-wide interactions of adult height on overall breast cancer risk. The genome-wide significance threshold of *P* < 5 x 10^−8^ is indicated by the dashed black line. Genome-wide significant findings are highlighted in blue

**Fig. 2:**
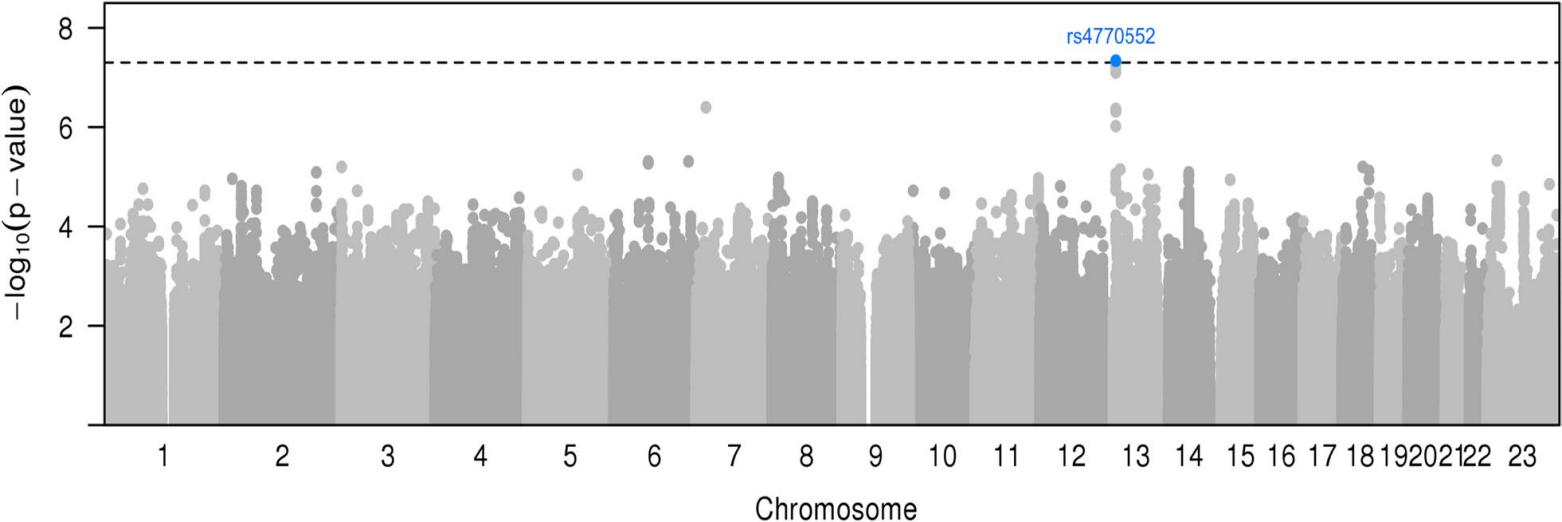
Manhattan plot of genome-wide interaction of age at menarche for ER + breast cancer risk. The genome-wide significance threshold of *P* < 5 x 10^−8^ is indicated by the dashed black line. Genome-wide significant findings are highlighted in blue.

The genome-wide analysis of interactions with seven environmental risk factors yielded two SNP-risk factor pairs at BFDP < 15%, one for risk of overall breast cancer and one for ER + breast cancer risk (Table 1, Fig. 1, 2, Additional file 1: Figure S1A-S1B). No inflation in the test statistics was observed for either of the environmental risk factors. The heritability on the frailty scale of breast cancer risk explained by G×E interaction is shown in Additional file 1: Figure S2. The estimated proportion of the frailty scale heritability explained by G×E interactions was very low for all factors, being highest for age at first full-term pregnancy (~ 1.5% for both overall and ER + breast cancer risk), age at menarche and post-menopausal BMI.

**Table 1:** Genetic variants with suggestive (P_int_ ≤ 5E−07) GxE interactions for overall and estrogen receptor positive (ER +) breast cancer risk

Risk factor	SNP	Chr	Position^1^	Alleles^2^	EAF^3^	OR_marg_ (95% CI)	P_marg_	OR_int_ (95% CI)	P_int_	BFDP^4^
*Overall breast cancer risk*										
Number of full-term pregnancies (per 1 birth)	rs10928872	2	129,833,111	A/T	0.08	1.00 (0.97–1.03)	0.97	0.93 (0.91–0.96)	1.34E-07	0.98
Number of full-term pregnancies (per 1 birth)	rs36064687	2	129,832,988	AT/A	0.08	1.00 (0.97–1.04)	0.99	0.93 (0.91–0.96)	1.34E-07	0.98
Number of full-term pregnancies (per 1 birth)	rs79929694	2	129,841,483	A/G	0.08	1.00 (0.96–1.03)	0.96	0.93 (0.91–0.96)	1.43E-07	0.98
Number of full-term pregnancies (per 1 birth)	rs77107485	2	129,843,663	T/G	0.08	1.00 (0.97–1.04)	0.89	0.93 (0.91–0.96)	2.79E-07	0.98
Number of full-term pregnancies (per 1 birth)	rs79722231	2	129,834,483	T/C	0.08	1.00 (0.97–1.04)	0.92	0.93 (0.91–0.96)	2.80E-07	0.98
Age at menarche (per 2 years)	rs73277506	7	21,112,413	C/T	0.02	1.03 (0.96–1.10)	0.39	1.26 (1.16–1.38)	1.46E-07	0.75
Current smoking (yes/no)	rs11322161	8	105,120,195	GC/G	0.29	1.00 (0.98–1.02)	0.63	1.19 (1.12–1.27)	9.26E-08	0.49
Adult height (per 5 cm)	rs80018847	9	28,326,896	A/G	0.14	1.00 (0.98–1.03)	0.88	0.94 (0.92–0.96)	4.34E-08	0.11
Adult height (per 5 cm)	rs1360506	9	28,339,154	G/C	0.16	1.00 (0.97–1.03)	0.96	0.95 (0.93–0.98)	2.13E-07	1.00
Adult height (per 5 cm)	rs1237669	17	41,912,024	T/G	0.91	1.01 (0.98–1.04)	0.62	0.94 (0.91–0.96)	5.00E-07	0.16
OC use (yes/no)	rs147290549	18	7,713,860	C/T	0.50	1.01 (0.99–1.02)	0.23	1.10 (1.06–1.14)	4.55E-07	0.59
OC use (yes/no)	rs664040	18	7,716,250	C/A	0.50	1.01 (0.99–1.02)	0.22	1.10 (1.06–1.14)	4.93E-07	0.59
Current smoking (yes/no)	rs75489324	21	26,302,665	G/C	0.02	0.98 (0.92–1.05)	0.87	0.58 (0.47–0.72)	4.58E-07	0.94
***ER**** + breast cancer risk*										
Age at menarche (per 2 years)	rs73277506	7	21,112,413	C/T	0.02	1.04 (0.97–1.12)	0.25	1.28 (1.17–1.41)	3.94E-07	0.76
Current smoking (yes/no)	rs11322161	8	105,120,195	GC/G	0.29	1.00 (0.98–1.02)	0.95	1.20 (1.12–1.29)	4.32E-07	0.81
Age at menarche (per 2 years)	rs4770552	13	24,594,430	C/T	0.86	1.02 (1.00–1.05)	0.10	0.91 (0.88–0.94)	4.62E-08	0.11
Age at menarche (per 2 years)	rs113684695	13	24,600,947	ACCTCGT GATCCGC/A	0.86	1.02 (1.00–1.05)	0.11	0.91 (0.88–0.94)	5.48E-08	0.11
Age at menarche (per 2 years)	rs9510997	13	24,592,857	G/T	0.86	1.02 (1.00–1.05)	0.10	0.91 (0.88–0.94)	6.26E-08	0.11
Age at menarche (per 2 years)	rs7321200	13	24,593,519	A/G	0.87	1.01 (0.99–1.04)	0.33	0.91 (0.87–0.94)	7.92E-08	0.11
Age at menarche (per 2 years)	rs4770553	13	24,595,995	C/T	0.85	1.02 (0.99–1.04)	0.22	0.92 (0.89–0.95)	4.31E-07	0.76
Age at menarche (per 2 years)	rs9551041	13	24,601,810	A/G	0.85	1.02 (0.99–1.04)	0.21	0.92 (0.89–0.95)	4.58E-07	0.76
Age at menarche (per 2 years)	rs1886805	13	24,602,494	G/A	0.85	1.02 (0.99–1.04)	0.21	0.92 (0.89–0.95)	4.62E-07	0.76
Age at menarche (per 2 years)	rs4770555	13	24,601,751	T/C	0.85	1.02 (0.99–1.04)	0.21	0.92 (0.89–0.95)	4.63E-07	0.76
Age at menarche (per 2 years)	rs4770556	13	24,601,760	A/G	0.85	1.02 (0.99–1.04)	0.21	0.92 (0.89–0.95)	4.68E-07	0.76
Age at menarche (per 2 years)	rs1886804	13	24,602,174	T/G	0.85	1.02 (0.99–1.04)	0.21	0.92 (0.89–0.95)	4.72E-07	0.76
Age at menarche (per 2 years)	rs1886803	13	24,602,165	T/C	0.85	1.02 (0.99–1.04)	0.21	0.92 (0.89–0.95)	4.72E-07	0.76
Age at menarche (per 2 years)	rs9553145	13	24,602,061	G/C	0.85	1.02 (0.99–1.04)	0.21	0.92 (0.89–0.95)	4.75E-07	0.76
Age at menarche (per 2 years)	rs4769305	13	24,592,448	T/C	0.85	1.02 (0.99–1.05)	0.17	0.92 (0.89–0.95)	4.76E-07	0.76
Age at menarche (per 2 years)	rs9553144	13	24,601,943	C/G	0.85	1.02 (0.99–1.04)	0.21	0.92 (0.89–0.95)	4.78E-07	0.76
Number of full-term pregnancies (per 1 birth)	rs930198	23	79,492,077	T/C	0.24	1.00 (0.98–1.02)	0.93	0.96 (0.94–0.97)	3.10E-07	0.99

SNP: Single Nucleotide Polymorphism; Chr: Chromosome; Ref: Reference Allele; Alt: Alternative Allele; EAF: Effect Allele Frequency; OR_marg_: SNP Marginal Odds ratio; CI: Confidence Interval; P_marg_: Marginal p-value (meta-analyzed); OR_int_: Interaction p-value (meta-analyzed); P_int_: Interaction p-value; BFDP: Bayesian False Discovery Probability; ABF: Approximate Bayes Probability; OC use: Ever use of oral contraceptives; ER + : Estrogen receptor positive breast cancer risk

^1^Build 37 Position

^2^Reference/Alternate alleles in Europeans (forward strand)

^3^Effect allele frequency in controls in OncoArray dataset

^4^Bayesian False Discovery Probability at prior probability of 1 × 10^–05^

For overall breast cancer risk, there was evidence of interaction between SNP rs80018847 and adult height (OR_int_ = 0.94, 95% CI 0.92–0.96, P_int_ = 4.34E−08, BFDP = 11%) without an apparent marginal effect of the rs80018847 variant (OR_marg_ = 1.00, 95% CI 0.98–1.03, P_marg_ = 0.88). By categories of adult height defined a priori, the estimated per allele OR_meta_ of rs80018847-G varied from 1.03 (95% CI 0.94–1.13, P_meta_ = 0.53) for women shorter than 158 cm, 1.13 (1.02–1.25) for women 158–162 cm in height, to OR_meta_ of 1.01 (95% CI 0.93–1.09, P_meta_ = 0.88) for women who were 168 cm or taller risk (Additional file 1: Table S4). Therefore, there is no linear relationship between the SNP and categories of adult height. The interaction with height was also observed for ER + breast cancer (OR_int_ 0.95, 95% CI 0.93–0.97, P_int_ = 5.62E-06) but not for ER negative (ER-) breast cancer risk (OR_int_ = 0.98, 95% CI 0.93–1.03, P_int_ = 0.77). The regional plot for overall breast cancer shows another SNP (rs1360506) at this locus in high linkage disequilibrium (LD) (r^2^ = 0.81) with rs80018847 (Additional file 1: Figure S3).

For risk of ER + breast cancer, a statistically significant interaction was observed between SNP rs4770552 and age at menarche (OR_int_ = 0.91, 95% CI 0.88–0.94, P_int_ = 4.62E−08, BFDP = 11%). There was weak evidence for a marginal association between the rs4770552-T allele and ER + breast cancer (OR_marg_ = 1.02, 95% CI 1.00–1.05, P_marg_ = 0.10). The per allele OR_meta_ appeared to decrease with increasing age at menarche, from 1.07 (95% CI 1.00–1.15, P_meta_ = 0.04) for age at menarche less than 13 years to 0.92 (95% CI 0.77–1.09, P_meta_ = 0.33) for age at menarche greater than 15 years (Additional file 1: Table S4). There was weaker evidence of interaction between SNP rs4770552 and age at menarche for overall breast cancer risk (OR_int_ = 0.93, 95% CI 0.90–0.96, P_int_ = 5.47E−06), but no interaction for ER- breast cancer risk (OR_int_ = 0.98, 95% CI 0.89–1.08), P_int_ = 0.66). At this locus, we found suggestive evidence of interactions between further 13 SNPs and age at menarche for ER + breast cancer risk. However, these 13 SNPs are in high LD (r^2^ = 0.8–1.0) with SNP rs4770552 (Additional file 1: Figure S4).

## Discussion

This is the largest genome-wide gene-environment interaction study for breast cancer to date. We found evidence of one novel susceptibility loci interacting with adult height associated with increased breast cancer risk overall, and one interaction for increased risk of ER + breast cancer with age at menarche. It is important to note, however, that while these associations reached conventional levels of genome-wide statistical significance, they may still represent chance associations. Based on the assumed prior distribution of effect sizes, the BFDP for both loci were 11%, considered noteworthy. Nevertheless, studies with an even larger sample size are required to confirm or refute these associations.

Many observational studies have shown an association between increasing adult height and increased breast cancer risk, in both premenopausal and postmenopausal women [7, 29, 30]. A meta-analysis estimated that each 10 cm increment in height was associated with a 17% increase in breast cancer risk [31]. The biological link between height and breast cancer is poorly understood, but some studies have suggested that increased height corresponds to more stem cells at risk of acquiring driver mutations [32]. Another hypothesis is that adult height could be a surrogate for nutritional intake, potentially implying a role for insulin-like growth factor 1 (*IGF1*) [33]. The functional basis of the potential interaction between adult height and the SNP rs80018847 is unclear. This SNP is in an intronic region of the leucine rich repeat and Ig domain containing 2 gene (*LINGO2)* on the short arm of chromosome 9 (9p13). This gene encodes a transmembrane protein belonging to the LINGO/LERN protein family [34]. Studies in mouse embryos have shown expression of *LINGO2* specifically in the central nervous system [34], but it has not been implicated in breast cancer to date.

Early age at menarche is known to be associated with elevated risk of breast cancer. There is an approximate 5% decrease in risk with each year delay in the initiation of menstruation [35]. It has been postulated that younger age at menarche corresponds to longer cumulative hormonal exposure and therefore elevated levels of estradiol [3, 36]. SNP rs4770552 is an intronic variant within the spermatogenesis associated 13 gene (*SPATA13*) at 13q12. *SPATA13* encodes a guanine nucleotide exchange factor (GEF) for RhoA, Rac1 and CDC42 GTPases [37, 38]. Although the role of this gene in breast cancer is still unclear, there could be an indirect link via the role of RhoA GTPases in breast tumorigenesis. Rho GTPase signaling is altered in human breast cancers, and dysregulation of Rho GTPase may have differential effects on the development of breast tumors depending on the stage and subtype [39]. Activation of RhoA results in release of megakaryoblastic leukemia 1 (*MKL1*), which in turn has been observed to alter the transcriptional activity of ERα, known to play a critical role in breast tumors [40]. Therefore, SNP rs4770552 may potentially indirectly interact with the regulatory region of *SPATA13* and affect the breast tumorigenesis process via activation of RHoA GTPases.

Given that the marginal effects of the common genetic variants are small and the associations of environmental risk factors with breast cancer are modest, interactions are also expected to be weak (Additional file 1: Figure S5). Although this is the largest breast cancer dataset available to date with more than 60,000 cases and 70,000 controls, the study is underpowered to detect weak interactions. Also, this study included only women of European ancestry and the findings may not be generalizable to women of other ancestries.

Using LDSC regression, we estimated the overall heritability due to G×E for each of the risk factors. The estimated frailty scale heritability (≤ 0.015) can be compared with corresponding heritability for the SNP main effects (for which heritability is about 0.47) or the overall heritability based on the familial risk (~ 1.4) [28, 41]. The implication is that G×E interactions make very little contribution to the heritability of breast cancer, at least for the known risk factors and common genetic variants that can be evaluated using genome-wide arrays, and hence do not make an important contribution to risk prediction at the population level. This is consistent with the fact that detection of G×E interactions is rare. This does not rule out the possibility that G×E interactions could be identified in additional large studies or that such interactions may provide important clues to mechanisms.

## Conclusions

In conclusion, we identified two novel genome-wide gene–environment interactions for overall and ER + breast cancer risk for women of European ancestry. These results contribute to our global body of knowledge on genetic susceptibility for breast cancer by generating plausible biological hypotheses, but they require replication and further functional studies.

### Supplementary Information

**Additional file 1**. A genome-wide gene-environment interaction study of breast cancer risk for women of European ancestry. **Supplementary Table 1:** Participating studies with number of total cases and controls per study. **Supplementary Table 2:** Detailed information of the characteristics of the study population by study design and case-control status. **Supplementary Table 3:** Associations of epidemiological risk factors for overall and ER-specific subtype breast cancer risk in population-based and cohort studies. **Supplementary Table 4:** Stratified analysis results for genome-wide significant interaction results by categories of risk factors. **Supplementary Figure 1:** Quantile-Quantile (Q-Q) plots of genome-wide interaction of A) Adult height on overall breast cancer risk and B) Age at menarche on ER+ breast cancer risk. **Supplementary Figure 2:** Frailty-scale heritability explained by GxE interaction on overall and estrogen receptor positive breast cancer risk. **Supplementary Figure 3:** Regional association plot for the interaction analyses between SNP rs80018847 and adult height for overall breast cancer risk. **Supplementary Figure 4:** Regional association plot for the interaction analyses between SNP rs4770552 and age at menarche for ER+ breast cancer risk. **Supplementary Figure 5:** Power (x-axis) to detect gene-environment interaction odds ratio (y-axis) at different minor allele frequencies (0.01 to 0.5: legend below) for 1:1 unmatched case-control study for different sample sizes (N = 40,000 to 120,000 with 10,000 increment). Power calculation was performed by Quanto 1.2.4, assuming a log additive model with SNP marginal effect estimate as 1.10, marginal effect estimate of the environmental risk factor as 1.20, and a two-side alpha of 5 x 10-08. We also assumed a 15% prevalence of the environmental risk factor and 1% prevalence of the disease.

## Data Availability

The datasets analyzed during the current study are not publicly available but are available upon request and approval of BCAC Data Access Co-ordinating Committee.

## Abbreviations

BMI: Body mass index
COGS: Collaborative Oncological Gene-Environment Study
SNPs: Single nucleotide polymorphisms
GWAS: Genome-wide association study
GEWIS: Genome-wide gene-environment interaction study
G×E: Gene–environment interaction
BCAC: Breast Cancer Association Consortium
ER +: Estrogen receptor positive
ER-: Estrogen receptor negative
Q–Q: Quantile–quantile
BFDP: Bayesian false discovery probability
MAF: Minor allele frequency
OR_int_: Interaction odds ratio
CI: Confidence intervals
OR_meta_: Meta-analyzed odds ratio
LD: Linkage disequilibrium
IGF1: Insulin-like growth factor 1
LINGO2: Leucine rich repeat and Ig domain containing 2
SPATA13: Spermatogenesis associated 13
MKL1: Megakaryoblastic leukemia 1
LDSC: Linkage disequilirium score regression
