# Supplementary material for: A genome-wide gene-environment interaction study of breast cancer risk for women of European ancestry
Source: Breast Cancer Res. 2023 Aug 9;25:93. doi: 10.1186/s13058-023-01691-8 (PMC10411002; doi:10.1186/s13058-023-01691-8)

**A genome-wide gene-environment interaction study of breast cancer risk for women of European ancestry**

Pooja Middha^1*^, Xiaoliang Wang^2, 3^, Sabine Behrens^1^, Manjeet K. Bolla^4^, Qin Wang^4^, Joe Dennis^4^, Kyriaki Michailidou^4, 5^, Thomas U. Ahearn^6^, Irene L. Andrulis^7, 8^, Hoda Anton-Culver^9^, Volker Arndt^10^, Kristan J. Aronson^11^, Paul L. Auer^12^, Annelie Augustinsson^13^, Thaïs Baert^14^, Laura E. Beane Freeman^6^, Heiko Becher^15^, Matthias W. Beckmann^16^, Javier Benitez^17, 18^, Stig E. Bojesen^19-21^, Hiltrud Brauch^22-24^, Hermann Brenner^10, 25, 26^, Angela Brooks-Wilson^27^, Daniele Campa^1, 28^, Federico Canzian^29^, Angel Carracedo^30, 31^, Jose E. Castelao^32^, Stephen J. Chanock^6^, Georgia Chenevix-Trench^33^, CTS Consortium^34, 35^, Emilie Cordina-Duverger^36^, Fergus J. Couch^37^, Angela Cox^38^, Simon S. Cross^39^, Kamila Czene^40^, Laure Dossus^41^, Pierre-Antoine Dugué^42, 43^, A. Heather Eliassen ^44-46^, Mikael Eriksson^40^, D. Gareth Evans^47, 48^, Peter A. Fasching^16^, Jonine D. Figueroa^6, 49, 50^, Olivia Fletcher^51^, Henrik Flyger^52^, Marike Gabrielson^40^, Manuela Gago-Dominguez^30^, Graham G. Giles^42, 43, 53^, Anna González-Neira^54^, Felix Grassmann^40, 55^, Anne Grundy^56^, Pascal Guénel^36^, Christopher A. Haiman^57^, Niclas Håkansson^58^, Per Hall^40, 59^, Ute Hamann^60^, Susan E. Hankinson^44, 61^, Elaine F. Harkness^62-64^, Bernd Holleczek^65^, Reiner Hoppe^22, 66^, John L. Hopper^53^, Richard S. Houlston^67^, Anthony Howell^68^, David J. Hunter^45, 69^, Christian Ingvar^70^, ABCTB Investigators^71^, kConFab Investigators^72, 73^, Karolin Isaksson^74^, Helena Jernström^13^, Esther M. John^75, 76^, Michael E. Jones^67^, Rudolf Kaaks^1^, Renske Keeman^77^, Cari M. Kitahara^78^, Yon-Dschun Ko^79^, Stella Koutros^6^, Allison W. Kurian^75, 76^, James V. Lacey^34, 35^, Diether Lambrechts^80, 81^, Nicole L. Larson^82^, Susanna Larsson^58, 83^, Loic Le Marchand^84^, Flavio Lejbkowicz^85^, Shuai Li^4, 42, 53^, Martha Linet^78^, Jolanta Lissowska^86^, Maria Elena Martinez^87^, Tabea Maurer^88^, Anna Marie Mulligan^89, 90^, Claire Mulot^91^, Rachel A. Murphy^92, 93^, William G. Newman^47, 48^, Sune F. Nielsen^19, 20^, Børge G. Nordestgaard^19-21^, Aaron Norman^82^, Katie M. O'Brien^94^, Janet E. Olson^82^, Alpa V. Patel^95^, Ross Prentice^96^, Erika Rees-Punia^95^, Gad Rennert^85^, Valerie Rhenius^97^, Kathryn J. Ruddy^98^, Dale P. Sandler^94^, Christopher G. Scott^99^, Mitul Shah^97^, Xiao-Ou Shu^100^, Ann Smeets^101^, Melissa C. Southey^42, 43, 102^, Jennifer Stone^53, 103^, Rulla M. Tamimi^45, 104^, Jack A. Taylor^94, 105^, Lauren R. Teras^95^, Katarzyna Tomczyk^51^, Melissa A. Troester^106^, Thérèse Truong^36^, Celine M. Vachon^82^, Sophia S. Wang^34, 35^, Clarice R. Weinberg^107^, Hans Wildiers^14^, Walter Willett ^44-46^, Stacey J. Winham^108^, Alicja Wolk^58, 83^, Xiaohong R. Yang^6^, M. Pilar Zamora^109^, Wei Zheng^100^, Argyrios Ziogas^9^, Alison M. Dunning^97^, Paul D.P. Pharoah^4, 97^, Montserrat García-Closas^6^, Marjanka K. Schmidt^77, 110^, Peter Kraft^45, 111^, Roger L. Milne^42, 43, 53^, Sara Lindström^2, 3^, Douglas F. Easton^4, 97#^, Jenny Chang-Claude^1, 88#^.

^1^ Division of Cancer Epidemiology, German Cancer Research Center (DKFZ), Heidelberg, Germany.

^2^ Department of Epidemiology, University of Washington School of Public Health, Seattle, WA, USA.

^3^ Public Health Sciences Division, Fred Hutchinson Cancer Research Center, Seattle, WA, USA.

^4^ Centre for Cancer Genetic Epidemiology, Department of Public Health and Primary Care, University of Cambridge, Cambridge, UK.

^5^ Biostatistics Unit, The Cyprus Institute of Neurology & Genetics, Nicosia, Cyprus.

^6^ Division of Cancer Epidemiology and Genetics, National Cancer Institute, National Institutes of Health, Department of Health and Human Services, Bethesda, MD, USA.

^7^ Fred A. Litwin Center for Cancer Genetics, Lunenfeld-Tanenbaum Research Institute of Mount Sinai Hospital, Toronto, Ontario, Canada.

^8^ Department of Molecular Genetics, University of Toronto, Toronto, Ontario, Canada.

^9^ Department of Medicine, Genetic Epidemiology Research Institute, University of California Irvine, Irvine, CA, USA.

^10^ Division of Clinical Epidemiology and Aging Research, German Cancer Research Center (DKFZ), Heidelberg, Germany.

^11^ Department of Public Health Sciences, and Cancer Research Institute, Queen’s University, Kingston, ON, Canada.

^12^ Division of Biostatistics, Institute for Health and Equity, and Cancer Center, Medical College of Wisconsin, Milwaukee, WI, USA.

^13^ Oncology, Clinical Sciences in Lund, Lund University, Lund, Sweden.

^14^ Leuven Multidisciplinary Breast Center, Department of Oncology, Leuven Cancer Institute, University Hospitals Leuven, Leuven, Belgium.

^15^ Institute of Medical Biometry and Epidemiology, University Medical Center Hamburg-Eppendorf, Hamburg, Germany.

^16^ Department of Gynecology and Obstetrics, Comprehensive Cancer Center Erlangen-EMN, Friedrich-Alexander University Erlangen-Nuremberg, University Hospital Erlangen, Erlangen, Germany.

^17^ Human Genetics Group, Spanish National Cancer Research Centre (CNIO), Madrid, Spain.

^18^ Centre for Biomedical Network Research on Rare Diseases (CIBERER), Instituto de Salud Carlos III, Madrid, Spain.

^19^ Copenhagen General Population Study, Herlev and Gentofte Hospital, Copenhagen University Hospital, Herlev, Denmark.

^20^ Department of Clinical Biochemistry, Herlev and Gentofte Hospital, Copenhagen University Hospital, Herlev, Denmark.

^21^ Faculty of Health and Medical Sciences, University of Copenhagen, Copenhagen, Denmark.

^22^ Dr. Margarete Fischer-Bosch-Institute of Clinical Pharmacology, Stuttgart, Germany.

^23^ iFIT-Cluster of Excellence, University of Tübingen, Tübingen, Germany.

^24^ German Cancer Consortium (DKTK) and German Cancer Research Center (DKFZ), Partner Site Tübingen, Tübingen, Germany.

^25^ Division of Preventive Oncology, German Cancer Research Center (DKFZ) and National Center for Tumor Diseases (NCT), Heidelberg, Germany.

^26^ German Cancer Consortium (DKTK), German Cancer Research Center (DKFZ), Heidelberg, Germany.

^27^ Canada's Michael Smith Genome Sciences Centre, BC Cancer, Vancouver, BC, Canada.

^28^ Department of Biology, University of Pisa, Pisa, Italy.

^29^ Genomic Epidemiology Group, German Cancer Research Center (DKFZ), Heidelberg, Germany.

^30^ Genomic Medicine Group, International Cancer Genetics and Epidemiology Group, Fundación Pública Galega de Medicina Xenómica, Instituto de Investigación Sanitaria de Santiago de Compostela (IDIS), Complejo Hospitalario Universitario de Santiago, SERGAS, Santiago de Compostela, Spain.

^31^ Grupo de Medicina Xenómica, Centro de Investigación en Red de Enfermedades Raras (CIBERER) y Centro Nacional de Genotipado (CEGEN-PRB2), Universidad de Santiago de Compostela, Santiago de Compostela, Spain.

^32^ Oncology and Genetics Unit, Instituto de Investigación Sanitaria Galicia Sur (IISGS), Xerencia de Xestion Integrada de Vigo-SERGAS, Vigo, Spain.

^33^ Department of Genetics and Computational Biology, QIMR Berghofer Medical Research Institute, Brisbane, Queensland, Australia.

^34^ Department of Computational and Quantitative Medicine, City of Hope, Duarte, CA, USA.

^35^ City of Hope Comprehensive Cancer Center, City of Hope, Duarte, CA, USA.

^36^ Team 'Exposome and Heredity', CESP, Gustave Roussy, INSERM, University Paris-Saclay, UVSQ, Villejuif, France.

^37^ Department of Laboratory Medicine and Pathology, Mayo Clinic, Rochester, MN, USA.

^38^ Sheffield Institute for Nucleic Acids (SInFoNiA), Department of Oncology and Metabolism, University of Sheffield, Sheffield, UK.

^39^ Academic Unit of Pathology, Department of Neuroscience, University of Sheffield, Sheffield, UK.

^40^ Department of Medical Epidemiology and Biostatistics, Karolinska Institutet, Stockholm, Sweden.

^41^ Nutrition and Metabolism Section, International Agency for Research on Cancer (IARC-WHO), Lyon, France.

^42^ Precision Medicine, School of Clinical Sciences at Monash Health, Monash University, Clayton, Victoria, Australia.

^43^ Cancer Epidemiology Division, Cancer Council Victoria, Melbourne, Victoria, Australia.

^44^ Channing Division of Network Medicine, Department of Medicine, Brigham and Women's Hospital and Harvard Medical School, Boston, MA, USA.

^45^ Department of Epidemiology, Harvard T.H. Chan School of Public Health, Boston, MA, USA.

^46^ Department of Nutrition, Harvard T.H. Chan School of Public Health, Boston, MA, USA.

^47^ Division of Evolution and Genomic Sciences, School of Biological Sciences, Faculty of Biology, Medicine and Health, University of Manchester, Manchester Academic Health Science Centre, Manchester, UK.

^48^ North West Genomics Laboratory Hub, Manchester Centre for Genomic Medicine, St Mary’s Hospital, Manchester University NHS Foundation Trust, Manchester Academic Health Science Centre, Manchester, UK.

^49^ Usher Institute of Population Health Sciences and Informatics, The University of Edinburgh, Edinburgh, UK.

^50^ Cancer Research UK Edinburgh Centre, The University of Edinburgh, Edinburgh, UK.

^51^ The Breast Cancer Now Toby Robins Research Centre, The Institute of Cancer Research, London, UK.

^52^ Department of Breast Surgery, Herlev and Gentofte Hospital, Copenhagen University Hospital, Herlev, Denmark.

^53^ Centre for Epidemiology and Biostatistics, Melbourne School of Population and Global Health, The University of Melbourne, Melbourne, Victoria, Australia.

^54^ Human Cancer Genetics Programme, Spanish National Cancer Research Centre (CNIO), Madrid, Spain.

^55^ Institute for Clinical Research and Systems Medicine, Health and Medical University, Potsdam, Germany.

^56^ Department of Public Health Sciences, Queen's University, Kingston, Ontario, Canada.

^57^ Department of Preventive Medicine, Keck School of Medicine, University of Southern California, Los Angeles, CA, USA.

^58^ Institute of Environmental Medicine, Karolinska Institutet, Stockholm, Sweden.

^59^ Department of Oncology, Södersjukhuset, Stockholm, Sweden.

^60^ Molecular Genetics of Breast Cancer, German Cancer Research Center (DKFZ), Heidelberg, Germany.

^61^ Department of Biostatistics & Epidemiology, University of Massachusetts, Amherst, Amherst, MA, USA.

^62^ Division of Informatics, Imaging and Data Sciences, Faculty of Biology, Medicine and Health, University of Manchester, Manchester Academic Health Science Centre, Manchester, UK.

^63^ Nightingale & Genesis Prevention Centre, Wythenshawe Hospital, Manchester University NHS Foundation Trust, Manchester, UK.

^64^ NIHR Manchester Biomedical Research Unit, Manchester University NHS Foundation Trust, Manchester Academic Health Science Centre, Manchester, UK.

^65^ Saarland Cancer Registry, Saarbrücken, Germany.

^66^ University of Tübingen, Tübingen, Germany.

^67^ Division of Genetics and Epidemiology, The Institute of Cancer Research, London, UK.

^68^ Division of Cancer Sciences, University of Manchester, Manchester, UK.

^69^ Nuffield Department of Population Health, University of Oxford, Oxford, UK.

^70^ Surgery, Clinical Sciences in Lund, Lund University, Lund, Sweden.

^71^ Australian Breast Cancer Tissue Bank, Westmead Institute for Medical Research, University of Sydney, Sydney, New South Wales, Australia.

^72^ Research Department, Peter MacCallum Cancer Center, Melbourne, Victoria, Australia.

^73^ Sir Peter MacCallum Department of Oncology, The University of Melbourne, Melbourne, Victoria, Australia.

^74^ Surgery, Clinical Sciences in Lund, Lund University, Lund & Department of Surgery, Kristianstad Hospital, Kristianstad, Sweden.

^75^ Department of Epidemiology and Population Health, Stanford University School of Medicine, Stanford, CA, USA.

^76^ Department of Medicine, Division of Oncology, Stanford Cancer Institute, Stanford University School of Medicine, Stanford, CA, USA.

^77^ Division of Molecular Pathology, The Netherlands Cancer Institute, Amsterdam, the Netherlands.

^78^ Radiation Epidemiology Branch, Division of Cancer Epidemiology and Genetics, National Cancer Institute, Bethesda, MD, USA.

^79^ Department of Internal Medicine, Johanniter GmbH Bonn, Johanniter Krankenhaus, Bonn, Germany.

^80^ Laboratory for Translational Genetics, Department of Human Genetics, KU Leuven, Leuven, Belgium.

^81^ VIB Center for Cancer Biology, VIB, Leuven, Belgium.

^82^ Department of Quantitative Health Sciences, Division of Epidemiology, Mayo Clinic, Rochester, MN, USA.

^83^ Department of Surgical Sciences, Uppsala University, Uppsala, Sweden.

^84^ Epidemiology Program, University of Hawaii Cancer Center, Honolulu, HI, USA.

^85^ Clalit National Cancer Control Center, Carmel Medical Center and Technion Faculty of Medicine, Haifa, Israel.

^86^ Department of Cancer Epidemiology and Prevention, M. Sklodowska-Curie National Research Oncology Institute, Warsaw, Poland.

^87^ Moores Cancer Center and Herbert Wertheim School of Public Health and Human Longevity Science, University of California, San Diego, La Jolla, CA, USA.

^88^ Cancer Epidemiology Group, University Cancer Center Hamburg (UCCH), University Medical Center Hamburg-Eppendorf, Hamburg, Germany.

^89^ Department of Laboratory Medicine and Pathobiology, University of Toronto, Toronto, Ontario, Canada.

^90^ Laboratory Medicine Program, University Health Network, Toronto, Ontario, Canada.

^91^ Université Paris Cité, INSERM UMR-S1138. CRB EPIGENETEC, Paris, France.

^92^ School of Population and Public Health, University of British Columbia, Vancouver, BC, Canada.

^93^ Cancer Control Research, BC Cancer, Vancouver, BC, Canada.

^94^ Epidemiology Branch, National Institute of Environmental Health Sciences, NIH, Research Triangle Park, NC, USA.

^95^ Department of Population Science, American Cancer Society, Atlanta, GA, USA.

^96^ Cancer Prevention Program, Fred Hutchinson Cancer Research Center, Seattle, WA, USA.

^97^ Centre for Cancer Genetic Epidemiology, Department of Oncology, University of Cambridge, Cambridge, UK.

^98^ Department of Oncology, Mayo Clinic, Rochester, MN, USA.

^99^ Department of Quantitative Health Sciences, Division of Clinical Trials and Biostatistics, Mayo Clinic, Rochester, MN, USA.

^100^ Division of Epidemiology, Department of Medicine, Vanderbilt Epidemiology Center, Vanderbilt-Ingram Cancer Center, Vanderbilt University School of Medicine, Nashville, TN, USA.

^101^ Department of Surgical Oncology, University Hospitals Leuven, Leuven, Belgium.

^102^ Department of Clinical Pathology, The University of Melbourne, Melbourne, Victoria, Australia.

^103^ Genetic Epidemiology Group, School of Population and Global Health, University of Western Australia, Perth, Western Australia, Australia.

^104^ Department of Population Health Sciences, Weill Cornell Medicine, New York, NY, USA.

^105^ Epigenetic and Stem Cell Biology Laboratory, National Institute of Environmental Health Sciences, NIH, Research Triangle Park, NC, USA.

^106^ Department of Epidemiology, Gillings School of Global Public Health and UNC Lineberger Comprehensive Cancer Center, University of North Carolina at Chapel Hill, Chapel Hill, NC, USA.

^107^ Biostatistics and Computational Biology Branch, National Institute of Environmental Health Sciences, NIH, Research Triangle Park, NC, USA.

^108^ Department of Quantitative Health Sciences, Division of Computational Biology, Mayo Clinic, Rochester, MN, USA.

^109^ Servicio de Oncología Médica, Hospital Universitario La Paz, Madrid, Spain.

^110^ Division of Psychosocial Research and Epidemiology, The Netherlands Cancer Institute - Antoni van Leeuwenhoek hospital, Amsterdam, the Netherlands.

^111^ Program in Genetic Epidemiology and Statistical Genetics, Harvard T.H. Chan School of Public Health, Boston, MA, USA.

^*^ Corresponding author

^#^ Joint senior authors

Corresponding Author:

Pooja Middha, PhD

1450 3^rd^ St., Rm 289

San Francisco, CA 94158

Email: pooja.middha@ucsf.edu

| Supplementary Table 1: Participating studies with number of total cases and controls per study | | | | | |
| --- | --- | --- | --- | --- | --- |
| *Study name* | *Study acronym* | *Country* | *Study Design^1^* | *Cases* | *Controls* |
| Australian Breast Cancer Family Study | ABCFS | Australia | Population-based case-control study | 1317 | 738 |
| Amsterdam Breast Cancer Study | ABCS | Netherlands | Non population-based study | 442 | 1376 |
| Australian Breast Cancer Tissue Bank | ABCTB | Australia | Non population-based study | 947 | 375 |
| Agricultural Health Study | AHS | USA | Prospective cohort study | 513 | 1137 |
| Bavarian Breast Cancer Cases and Controls | BBCC | Germany | Non population-based study | 809 | 706 |
| Breast Cancer Employment and Environment Study | BCEES | Australia | Population-based case-control study | 783 | 834 |
| Breast Cancer in Northern Israel Study | BCINIS | Israel | Population-based case-control study | 1315 | 724 |
| Breast Oncology Galicia Network | BREOGAN | Spain | Non population-based study | 1265 | 725 |
| Canadian Breast Cancer Study | CBCS | Canada | Population-based case-control study | 568 | 817 |
| CECILE Breast Cancer Study | CECILE | France | Population-based case-control study | 910 | 1002 |
| Copenhagen General Population Study | CGPS | Denmark | Non population-based study | 4064 | 5241 |
| Spanish National Cancer Centre Breast Cancer Study | CNIO-BCS | Spain | Non population-based study | 746 | 829 |
| Cancer Prevention Study-II Nutrition Cohort | CPSII | USA | Prospective cohort study | 2546 | 3323 |
| California Teachers Study | CTS | USA | Prospective cohort study | 1156 | 610 |
| European Prospective Investigation Into Cancer and Nutrition | EPIC | France, Germany, Greece, Italy, Spain, The Netherlands, and UK | Prospective cohort study | 3436 | 3597 |
| ESTHER Breast Cancer Study | ESTHER | Germany | Population-based case-control study | 476 | 505 |
| Gene Environment Interaction and Breast Cancer in Germany | GENICA | Germany | Population-based case-control study | 912 | 710 |
| Genetic Epidemiology Study of Breast Cancer by Age 50 | GESBC | Germany | Population-based case-control study | 316 | 181 |
| Karolinska Mammography Project for Risk Prediction of Breast Cancer - Cohort Study | KARMA | Sweden | Prospective cohort study | 1415 | 6026 |
| Kathleen Cuningham Foundation Consortium for research into Familial Breast Cancer/Australian Ovarian Cancer Study | KCONFAB/AOCS | Australia and New Zealand | Non population-based study | 251 | 896 |
| Leuven Multidisciplinary Breast Centre | LMBC | Belgium | Non population-based study | 3003 | 1821 |
| Mammary Carcinoma Risk Factor Investigation | MARIE | Germany | Population-based case-control study | 1643 | 2065 |
| Mayo Clinic Breast Cancer Study | MCBCS | USA | Non population-based study | 2062 | 2041 |
| Melbourne Collaborative Cohort Study | MCCS | Australia | Prospective cohort study | 1002 | 1206 |
| Multiethnic Cohort | MEC | USA | Prospective cohort study | 668 | 724 |
| Melanoma Inquiry of Southern Sweden | MISS | Sweden | Prospective cohort study | 599 | 1529 |
| Mayo Mammography Health Study | MMHS | USA | Prospective cohort study | 276 | 1635 |
| Nashville Breast Health Study | NBHS | USA | Population-based case-control study | 482 | 652 |
| Northern California Breast Cancer Family Registry | NC-BCFR | USA | Non population-based study | 696 | 150 |
| North Carolina Breast Cancer Study | NCBCS | USA | Population-based case-control study | 2074 | 1006 |
| Nurses' Health Study | NHS | USA | Prospective cohort study | 1103 | 1804 |
| Nurses' Health Study 2 | NHS2 | USA | Prospective cohort study | 1112 | 1905 |
| Ontario Familial Breast Cancer Registry | OFBCR | Canada | Non population-based study | 1934 | 728 |
| NCI Polish Breast Cancer Study | PBCS | Poland | Population-based case-control study | 1768 | 2082 |
| Karolinska Mammography Project for Risk Prediction of Breast Cancer - Case-Control Study | PKARMA | Sweden | Non population-based study | 3115 | 5464 |
| The Prostate, Lung, Colorectal and Ovarian Cancer Screening Trial | PLCO | USA | Prospective cohort study | 1822 | 2595 |
| Predicting the Risk Of Cancer At Screening Study | PROCAS | UK | Population-based case-control study | 342 | 1656 |
| Singapore and Sweden Breast Cancer Study | SASBAC | Sweden | Population-based case-control study | 1129 | 1373 |
| Sheffield Breast Cancer Study | SBCS | UK | Non population-based study | 594 | 848 |
| Study of Epidemiology and Risk factors in Cancer Heredity | SEARCH | UK | Non population-based study | 12571 | 8889 |
| The Sister Study | SISTER | USA | Prospective cohort study | 1501 | 1562 |
| Swedish Mammography Cohort | SMC | Sweden | Prospective cohort study | 1349 | 661 |
| UCI Breast Cancer Study | UCIBCS | USA | Non population-based study | 427 | 258 |
| UK Breakthrough Generations Study | UKBGS | UK | Prospective cohort study | 1047 | 1032 |
| US Radiologic Technologists Study | USRT | USA | Non population-based study | 848 | 1699 |
| Women's Health Initiative Observational Study | WHI | USA | Prospective cohort study | 4930 | 4617 |
| Total |  |  |  | 72284 | 80354 |
| ^1^Population-based design was defined as recruiting a random sample of all cases occurring in a geographically defined population during a specified period of time,and recruiting controls that were a random sample of the same source population as cases during the same period of time. Non-population-based design was defined as not strictly population-based (e.g. due to oversampling of selected participant groups for genotyping) or hospital-based. | | | | | |

| Supplementary Table 2: Detailed information of the characteristics of the study population by study design and case-control status | | | | | | | | |
| --- | --- | --- | --- | --- | --- | --- | --- | --- |
| *Characteristics* | *Population-based studies* | | | | *Non population-based studies* | | | |
|  | Cases  N (%) | Controls  N (%) | Cases  Mean (S.D.) | Controls  Mean (S.D.) | Cases  N (%) | Controls  N (%) | Cases  Mean (S.D.) | Controls  Mean (S.D.) |
| Reference age | 38510 | 48308 | 61.03  (11.27) | 58.66  (10.72) | 33774 | 32046 | 56.10  (11.39) | 54.80  (12.58) |
| ER status |  |  |  |  |  |  |  |  |
| Positive | 27830 (72.27) |  |  |  | 22385 (66.28) |  |  |  |
| Negative | 5783 (15.02) |  |  |  | 5113 (15.14) |  |  |  |
| Missing | 4897 (12.72) |  |  |  | 6276 (18.58) |  |  |  |
| Menopausal status |  |  |  |  |  |  |  |  |
| Premenopausal | 9045 (23.49) | 12047 (24.94) |  |  | 12556 (37.18) | 13424 (41.89) |  |  |
| Postmenopausal | 29465 (76.51) | 36261 (75.06) |  |  | 21218 (62.82) | 18622 (58.11) |  |  |
| Reproductive risk factors | | | | | | | | |
| Age at menarche (years) | 36893 | 46855 | 12.86  (1.53) | 12.96  (1.56) | 22415 | 19439 | 13.00  (1.57) | 12.99  (1.55) |
| Ever parous |  |  |  |  |  |  |  |  |
| Yes | 32025 (83.16) | 41555 (86.02) |  |  | 20442 (60.53) | 23398 (73.01) |  |  |
| No | 5217 (13.55) | 5618 (11.63) |  |  | 3933 (11.65) | 4127  (12.88) |  |  |
| Missing | 1268  (3.29) | 1135  (2.35) |  |  | 9399 (27.83) | 4521  (14.11) |  |  |
| Number of full-term pregnancies^a^ |  |  |  |  |  |  |  |  |
| 1 | 5572 (17.40) | 6182 (14.88) |  |  | 3912 (19.14) | 4151  (17.74) |  |  |
| 2 | 13004 (40.61) | 17091 (41.13) |  |  | 9811 (47.99) | 11263 (48.14) |  |  |
| 3 | 7735 (24.15) | 10427 (25.09) |  |  | 4511 (22.07) | 5140  (21.97) |  |  |
| ≥4 | 5323 (16.62) | 7652 (18.41) |  |  | 2048 (10.02) | 2146  (9.17) |  |  |
| Missing | 391  (1.22) | 203  (0.49) |  |  | 160  (0.78) | 698  (2.98) |  |  |
| Ever breastfed^a^ |  |  |  |  |  |  |  |  |
| Yes | 17358 (54.20) | 19953 (48.02) |  |  | 11298 (55.27) | 9543  (40.79) |  |  |
| No | 6155 (19.22) | 6557 (15.78) |  |  | 3167 (15.49) | 2409  (10.30) |  |  |
| Missing | 8512 (26.58) | 15045 (36.21) |  |  | 5977 (29.24) | 11446 (48.92) |  |  |
| Age at FFTP^a^ (years) | 30412 | 39987 | 24.92  (4.65) | 24.67  (4.54) | 17883 | 16192 | 25.10  (5.05) | 25.48  (4.82) |
| Anthropometric risk factors | | | | | | | | |
| Adult height (cm) | 35767 | 46506 | 163.58  (6.50) | 163.62  (6.50) | 23642 | 18359 | 164.13  (6.78) | 164.54  (6.88) |
| Postmenopausal BMI^c^ (kg/m^2^) | 28069 | 35112 | 26.52  (5.30) | 26.05  (4.98) | 14877 | 15508 | 26.45  (5.00) | 26.30  (4.86) |
| Hormonal risk factors | | | | | | | | |
| Ever use of oral contraceptives |  |  |  |  |  |  |  |  |
| Yes | 19632 (50.98) | 26311 (54.47) |  |  | 11018 (32.62) | 12356 (38.56) |  |  |
| No | 15750 (40.90) | 18441 (38.17) |  |  | 5080 (15.04) | 3419  (10.67) |  |  |
| Missing | 3128  (8.12) | 3556  (7.36) |  |  | 17676 (52.34) | 16271 (50.77) |  |  |
| Lifestyle risk factors | | | | | | | | |
| Current smoking |  |  |  |  |  |  |  |  |
| Yes | 4762 (12.37) | 5630 (11.65) |  |  | 2574  (7.62) | 2151  (6.71) |  |  |
| No | 28975 (75.24) | 37592 (77.82) |  |  | 11965 (35.43) | 15214 (47.48) |  |  |
| Missing | 4773 (12.39) | 5086 (10.53) |  |  | 19235 (56.95) | 14681 (45.81) |  |  |
| This table shows the number of cases and controls for each risk factor after all exclusions except for the exclusion of 150 cases and 150 controls for the variable of interest. This exclusion was conducted individually for each risk factor at the time of fitting logistic regression models. For continuous variables mean and standard deviation are reported, whereas, for categorical variables numbers and percentage are reported.  N: Number; %: Percentage; S.D.: Standard deviation; ER: Estrogen receptor; FFTP: First full-term pregnancy; BMI: Body mass index  ^a^ Among parous women, ^b^ Among premenopausal women, ^c^ Among postmenopausal women | | | | | | | | |

| Supplementary Table 3: Associations of epidemiological risk factors for overall and ER-specific subtype breast cancer risk in population-based and cohort studies | | | |
| --- | --- | --- | --- |
| *Environmental risk factor*^a^ | *Overall breast cancer risk*  *OR (95% CI)* | *ER-positive breast cancer risk*  *OR (95% CI)* | *ER-negative breast cancer risk*  *OR (95% CI)* |
|  |  |  |  |
| Age at menarche (per 2 years) | 0.91 (0.89-0.92) | 0.91 (0.89-0.93) | 0.89 (0.85-0.93) |
| Ever parous (yes/no) | 0.81 (0.77-0.84) | 0.78 (0.74-0.81) | 0.94 (0.85-1.04) |
| Number of full-term pregnancies (1,2,3,≥4)^1^ | 0.87 (0.85-0.88) | 0.86 (0.84-0.87) | 0.90 (0.86-0.94) |
| Age at first full-term pregnancy (per 5 years)^1^ | 1.14 (1.12-1.16) | 1.17 (1.14-1.19) | 1.02 (0.97-1.06) |
| Ever breastfed (yes/no)^1^ | 0.91 (0.88-0.95) | 0.92 (0.88-0.96) | 0.96 (0.88-1.03) |
| Adult height (per 5 cm) | 1.09 (1.08-1.10) | 1.10 (1.09-1.12) | 1.03 (1.00-1.05) |
| Postmenopausal BMI (per 5 kg/m^2^)^2^ | 1.07 (1.05-1.09) | 1.07 (1.05-1.09) | 1.05 (1.00-1.11) |
| Ever use of oral contraceptives (yes/no) | 1.22 (1.18-1.26) | 1.24 (1.20-1.29) | 1.14 (1.05-1.23) |
| Current smoking (yes/no)^3^ | 1.18 (1.13-1.24) | 1.18 (1.12-1.25) | 1.06 (0.96-1.18) |
| BMI: Body mass index | | | |
| ^a^All OR estimates are based on a model with single risk factor analyses adjusted for reference age and study | | | |
| ^1^ among parous women | | | |
| ^2^ among postmenopausal women | | | |
| ^3^ Additionally, model was adjusted for former smoking | | | |

| Supplementary Table 4: Stratified analysis results for genome-wide significant interaction results by categories of risk factors | | |
| --- | --- | --- |
| Categories of risk factor | OR  (95% CI) | P-value |
| *SNP rs80018847 – adult height (Overall Breast cancer risk)* | | |
| <158 cm | 1.03  (0.94-1.13) | 0.53 |
| 158 - < 162 cm | 1.13  (1.02-1.25) | 0.03 |
| 162 - <165 cm | 1.01  (0.91-1.12) | 0.89 |
| 165 - <168 cm | 1.07  (0.97-1.18) | 0.17 |
| ≥168 cm | 1.01  (0.93-1.09) | 0.88 |
|  |  |  |
| *SNP rs4770552 – age at menarche (ER+ Breast cancer risk)* | | |
| < 13 years | 1.07  (1.00-1.15) | 0.04 |
| 13 years | 0.98  (0.91-1.07) | 0.69 |
| 14 years | 0.98  (0.89-1.08) | 0.71 |
| 15 years | 0.83  (0.73-0.94) | 0.003 |
| >15 years | 0.92  (0.77-1.09) | 0.33 |
|  |  |  |

**Supplementary Figure 1: Quantile-Quantile (Q-Q) plots of genome-wide interaction of A) Adult height on overall breast cancer risk and B) Age at menarche on ER+ breast cancer risk**

1. Adult height on overall breast cancer risk


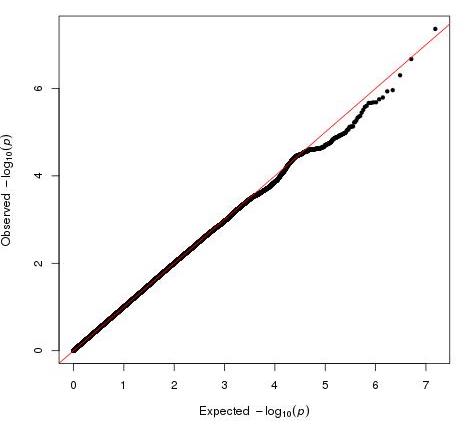


1. Age at menarche on ER+ breast cancer risk


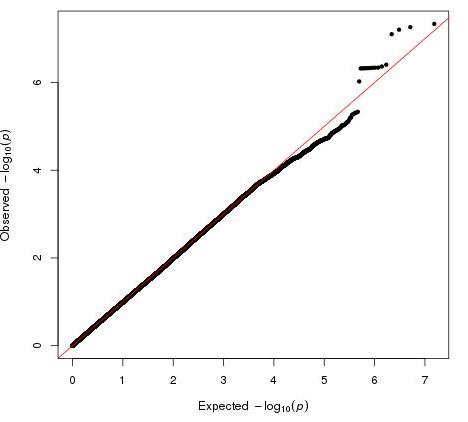


**Supplementary Figure 2: Frailty-scale heritability explained by GxE interaction on overall and estrogen receptor positive breast cancer risk.**


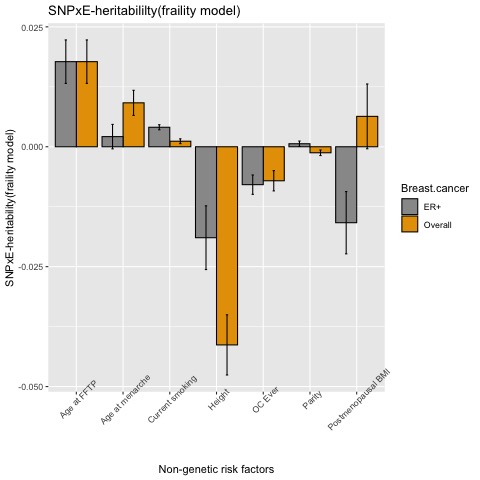


**Supplementary Figure 3: Regional association plot for the interaction analyses between SNP rs80018847 and adult height for overall breast cancer risk**


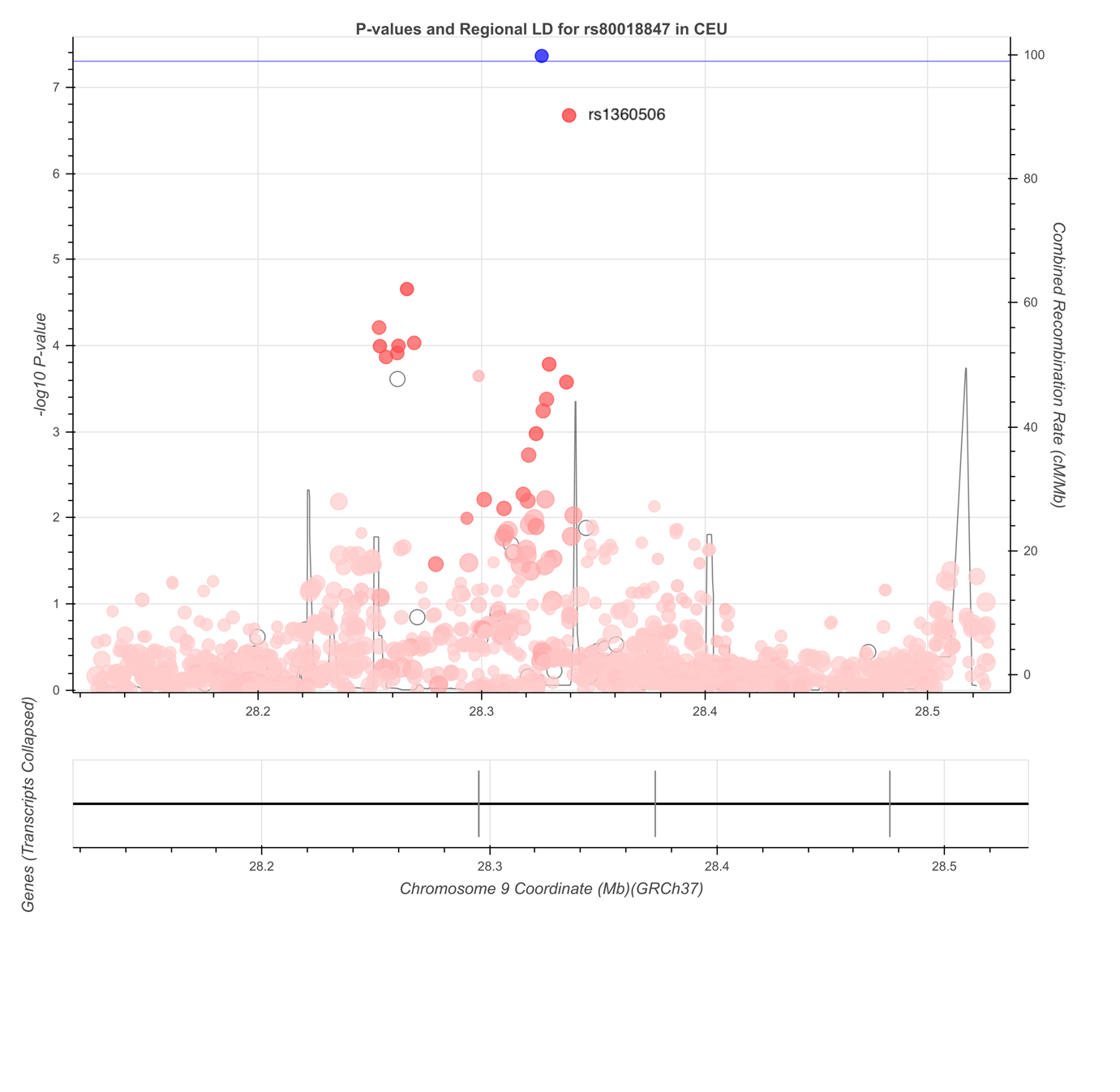


The -log10 of p-values (left y-axis) are plotted against the SNP genomic coordinates based on NCBI build 37 (x-axis). The estimated recombination rate from 1000 Genomes Project (European population) are on the right y-axis. SNP rs80018847 (the most significant SNP) is denoted in blue round. Darker the red, the higher the SNP in LD with the most significant SNP. The bottom panel shows gene annotations with collapsed transcripts. The gene annotations are from the UCSC browser.

**Supplementary Figure 4: Regional association plot for the interaction analyses between SNP rs4770552 and age at menarche for ER+ breast cancer risk**


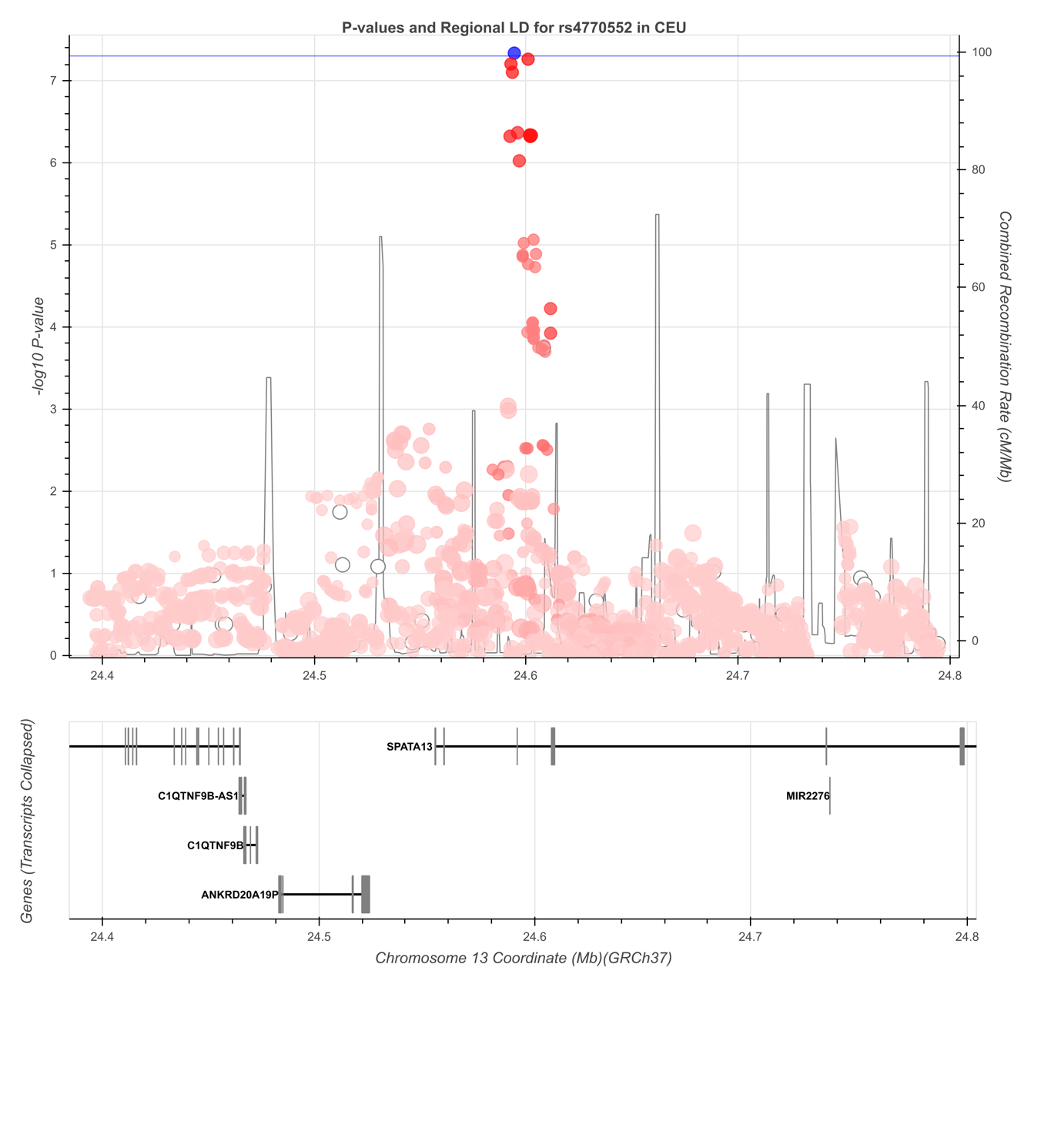


The -log10 of p-values (left y-axis) are plotted against the SNP genomic coordinates based on NCBI build 37 (x-axis). The estimated recombination rate from 1000 Genomes Project (European population) are on the right y-axis. SNP rs80018847 (the most significant SNP) is denoted in blue round. Darker the red, the higher the SNP in LD with the most significant SNP. The bottom panel shows gene annotations with collapsed transcripts. The gene annotations are from the UCSC browser.

**Supplementary Figure 5: Power (x-axis) to detect gene-environment interaction odds ratio (y-axis) at different minor allele frequencies (0.01 to 0.5: legend below) for 1:1 unmatched case-control study for different sample sizes (N = 40,000 to 120,000 with 10,000 increment). Power calculation was performed by Quanto 1.2.4, assuming a log-additive model with SNP marginal effect estimate as 1.10, marginal effect estimate of the environmental risk factor as 1.20, and a two-side alpha of 5 x 10^-08^. We also assumed a 15% prevalence of the environmental risk factor and 1% prevalence of the disease.**


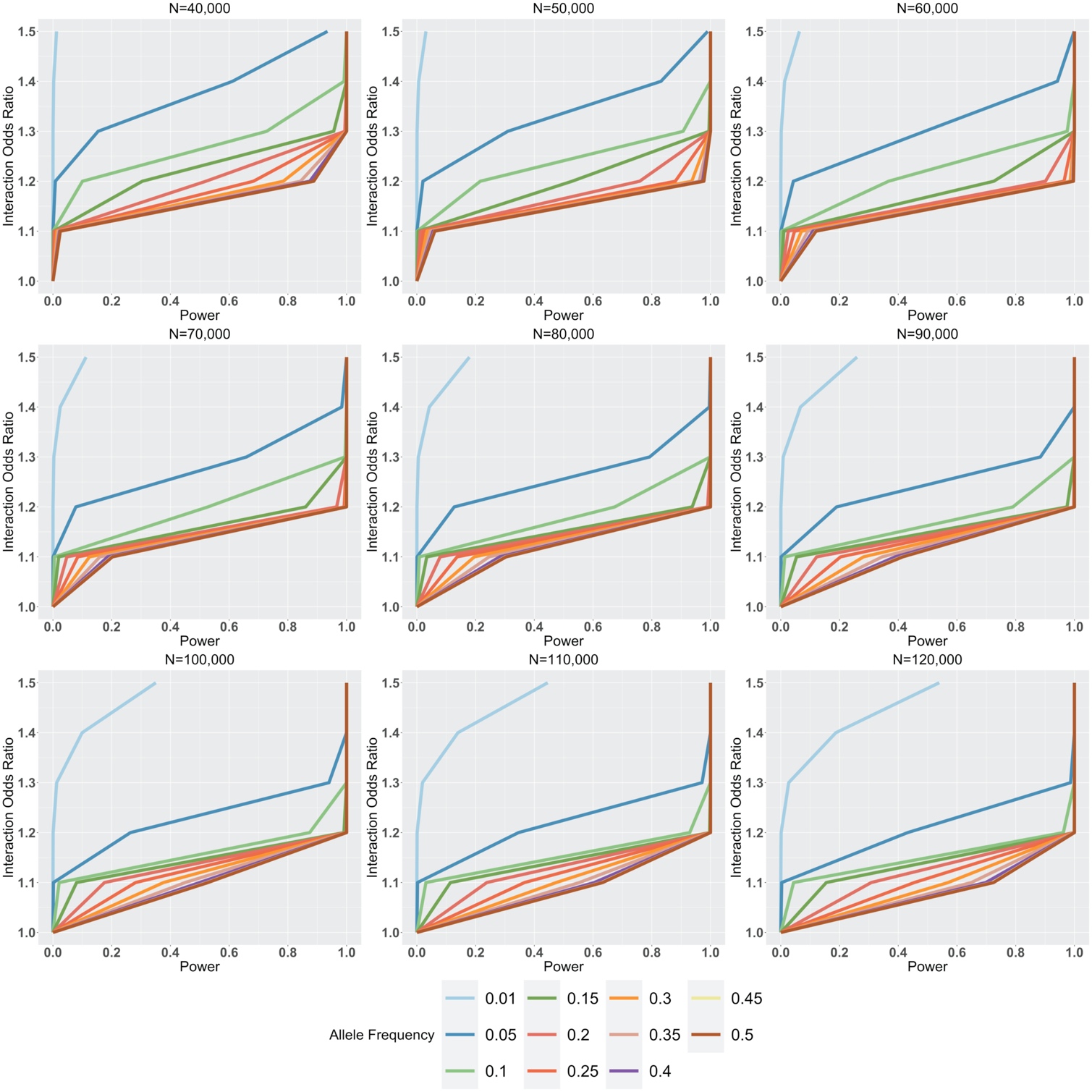

Supplement: Supplementary file 1 — Additional file 1. A genome-wide gene-environment interaction study of breast cancer risk for women of European ancestry. Supplementary Table 1: Participating studies with number of total cases and controls per study. Supplementary Table 2: Detailed information of the characteristics of the study population by study design and case-control status. Supplementary Table 3: Associations of epidemiological risk factors for overall and ER-specific subtype breast cancer risk in population-based and cohort studies. Supplementary Table 4: Stratified analysis results for genome-wide significant interaction results by categories of risk factors. Supplementary Figure 1: Quantile-Quantile (Q-Q) plots of genome-wide interaction of A) Adult height on overall breast cancer risk and B) Age at menarche on ER+ breast cancer risk. Supplementary Figure 2: Frailty-scale heritability explained by GxE interaction on overall and estrogen receptor positive breast cancer risk. Supplementary Figure 3: Regional association plot for the interaction analyses between SNP rs80018847 and adult height for overall breast cancer risk. Supplementary Figure 4: Regional association plot for the interaction analyses between SNP rs4770552 and age at menarche for ER+ breast cancer risk. Supplementary Figure 5: Power (x-axis) to detect gene-environment interaction odds ratio (y-axis) at different minor allele frequencies (0.01 to 0.5: legend below) for 1:1 unmatched case-control study for different sample sizes (N = 40,000 to 120,000 with 10,000 increment). Power calculation was performed by Quanto 1.2.4, assuming a log additive model with SNP marginal effect estimate as 1.10, marginal effect estimate of the environmental risk factor as 1.20, and a two-side alpha of 5 x 10^-08^. We also assumed a 15% prevalence of the environmental risk factor and 1% prevalence of the disease. [file 13058_2023_1691_MOESM1_ESM.docx]
